# Mouth Breathing and Orthodontic Referral in Pediatric Practice: A Cross-Sectional Survey

**DOI:** 10.3390/children12060790

**Published:** 2025-06-17

**Authors:** Tulca Büyükpatır Türk, Barış Erkut Türk, Yeşim Kaya

**Affiliations:** 1 Department of Orthodontics, Faculty of Dentistry, Ankara Yildirim Beyazit University, Ankara 06220, Türkiye; yesimkaya@aybu.edu.tr; 2 Department of Oral and Maxillofacial Surgery, Faculty of Dentistry, Gazi University, 06490 Ankara, Turkey; baristurk@gazi.edu.tr

**Keywords:** mouth breathing, pediatricians, maxillary constriction, orthodontic referral, awareness, interdisciplinary approach, early diagnosis, craniofacial development

## Abstract

**Objectives**: Mouth breathing in children may lead to craniofacial anomalies such as maxillary constriction. Pediatricians play a crucial role in recognizing early signs and making timely referrals. This study aimed to evaluate the awareness of pediatricians regarding mouth breathing and its orthodontic implications and to assess the impact of their treatment attitudes and clinical experience on referral practices. **Methods**: A 20-item online questionnaire was completed by 110 volunteer pediatricians from various regions of Türkiye via professional networks. The survey included items on awareness, treatment attitudes, referral behaviors, and demographics. Composite scores for awareness and orthodontic treatment attitude were calculated and included in the analysis. Data were analyzed using chi-square tests, Spearman correlation, and binary logistic regression. **Results**: Most pediatricians were aware of the link between mouth breathing and craniofacial issues (awareness rate: 73.6%), yet only 14.5% were familiar with specific orthodontic treatment approaches such as maxillary expansion. Although 70.9% expressed a desire for further training, only 25.5% reported frequently referring patients for orthodontic evaluation. Referral behavior was significantly associated with both clinical experience (*p* = 0.004) and orthodontic treatment attitude scores (*p* = 0.004) but not with awareness scores (*p* = 0.12). **Conclusions**: Although pediatricians in Türkiye demonstrate relatively high awareness regarding the consequences of mouth breathing, referral practices remain limited. Attitudinal orientation toward orthodontic treatment may play a more influential role in referral behavior than awareness alone.

## 1. Introduction

Mouth breathing is considered relatively common in pediatric populations and may often remain underdiagnosed. It frequently results from upper airway obstruction due to allergic rhinitis, adenoid or tonsillar hypertrophy, septal deviation, or chronic nasal inflammation [1,2,3,4]. Chronic mouth breathing may contribute to alterations in the physiological pattern of respiration, oral posture, and craniofacial development [3,5].

Clinical signs commonly associated with mouth breathing include an open lip posture, dry mouth, snoring, restless sleep, and reduced attention span during the day [1,5,6]. These functional disturbances have also been linked to broader impacts on pediatric health, including poorer sleep quality, behavioral difficulties, and potential influences on somatic growth and development [4,6]. These outcomes underscore the need for early recognition and a multidisciplinary approach to the management of children exhibiting mouth breathing.

In addition to these functional and behavioral impacts, mouth breathing may also affect craniofacial development. It has been associated with alterations in normal craniofacial growth patterns, contributing to dentofacial characteristics such as a narrow maxilla, increased lower anterior facial height, retrognathic mandible, anterior open bite, and characteristic “adenoid facies” [1,2,3,5,7]. These craniofacial changes may impact both facial aesthetics and upper airway function [3,7].

Given the potential for long-term skeletal and airway alterations, early and targeted treatment is critical. Effective management of mouth breathing requires addressing its underlying causes. Surgical interventions such as adenotonsillectomy may improve airway patency and positively influence facial growth patterns [8,9], while medical management of allergies and nasal inflammation remains essential. In addition, orthodontic interventions such as maxillary expansion may facilitate nasal airflow and support craniofacial development by increasing nasal cavity volume and addressing transverse maxillary deficiencies [3,8,10]. However, orthodontic therapy alone does not treat the underlying causes of mouth breathing and should be viewed as an adjunct to comprehensive medical and functional interventions [6,7,8,11].

Due to the multifactorial etiology of mouth breathing, a multidisciplinary approach is widely recognized as essential [4,6,7,11]. Pediatricians, as primary care providers for children, are typically the first to observe early signs of dysfunctional breathing and play a critical role in initiating diagnostic evaluation and coordinating referrals [4,7,11]. Effective management requires close collaboration among ear, nose, and throat (ENT) specialists, allergists, speech–language pathologists, and orthodontists to address both the functional and structural components of mouth breathing and to achieve comprehensive and lasting improvements in patient outcomes [4,6,7,11].

To our knowledge, no prior study has systematically evaluated pediatricians’ awareness of the orthodontic implications of mouth breathing or how their clinical attitudes may influence referral patterns. Previous research has suggested that pediatricians may have limited awareness or may not consistently refer affected children for orthodontic evaluation [12,13,14]. Therefore, this study aimed to assess pediatricians’ knowledge, attitudes, and referral practices regarding mouth breathing in children. It was hypothesized that higher levels of awareness and more favorable clinical attitudes would be associated with an increased likelihood of referral to orthodontists for children suspected of mouth breathing.

## 2. Materials and Methods

### 2.1. Survey

The sample size was calculated in the G*Power 3.1 program (Version 3.1 Franz Faul, Universität Kiel, Germany). Accordingly, an effect size of 0.8, a Type 1 and 2 error rate of 0.05, a confidence interval of 0.80, and a minimum number of participants of 90 were calculated.

The questionnaire was developed based on a comprehensive review of the literature on mouth breathing, pediatric screening behavior, and orthodontic referral patterns. It consisted of 20 items and was created by one pediatrician with at least 15 years of clinical experience and two orthodontists with over 10 years of experience each. Content validity of the questionnaire was established through a structured expert review process. One pediatrician and three orthodontists independently evaluated the draft questionnaire for clarity, relevance, and content coverage. Based on their feedback, ambiguous or inappropriate items were removed. Although no separate pilot testing or factor analysis was conducted, the final questionnaire was deemed sufficiently clear and comprehensive. Internal consistency of the awareness and attitude sections was subsequently evaluated via Cronbach’s alpha, which demonstrated acceptable reliability (awareness α = 0.658; attitude α = 0.787).

After obtaining ethical approval, the questionnaire was distributed online using Google Forms. The survey link was initially shared anonymously and voluntarily with pediatricians through professional WhatsApp groups (convenience sampling). The participants were also encouraged to forward the link to colleagues within their professional networks, creating a snowball sampling effect. A total of 113 responses were received; however, 3 were excluded due to incomplete responses. Thus, data from 110 pediatricians were included in the final analysis.

The questionnaire was divided into four sections. Questions 1–5 collected demographic information, including age, gender, years of experience, type of workplace, and number of patients seen per day. Questions 6–11 addressed awareness regarding mouth breathing and maxillary constriction. Questions 12–17 focused on attitudes toward orthodontic treatment and maxillary expansion, while questions 18–20 evaluated referral practices and interest in further education (Table 1). The questions were designed in multiple-choice format and included binary (“Yes”/“No”), ternary (e.g., “Yes”/“No”/“I do not have sufficient knowledge”), or broader categorical options depending on the content of the item.

Following ethical approval, the questionnaire was distributed online via Google Forms to anonymous volunteer pediatricians through professional WhatsApp groups. The participants were located in various regions of Türkiye, representing both urban and rural healthcare settings.

### 2.2. Statistical Analysis/Analytical Approach

Statistical analyses were performed using IBM SPSS Statistics (version 22, IBM Corp., Armonk, NY, USA). The normality of data distribution was assessed using visual methods, skewness, and kurtosis. Descriptive statistics were presented as frequencies, percentages, and medians where appropriate.

The internal consistency of the awareness (Q6–Q11) and attitude (Q12–Q17) subscales was evaluated using Cronbach’s alpha. The awareness subscale showed acceptable internal reliability (α = 0.658), while the attitude subscale demonstrated good reliability (α = 0.787). The referral-related items (Q18–Q20) exhibited poor internal consistency (α = –0.208) and were, therefore, analyzed individually rather than as a composite construct.

For inferential analysis, composite scores for awareness (Q6–Q11) and attitude (Q12–Q17) were calculated for each participant by summing binary-coded responses (Yes = 1, No/Unsure = 0) across each subscale (possible score range: 0–6 for each). These scores were treated as continuous variables in correlation and regression analyses. Additionally, participants were categorized into high or low awareness and attitude groups using a cutoff score of ≥5 for certain analyses, allowing for both linear and categorical effects to be assessed.

Chi-square tests were applied to individual item responses and demographic variables to explore item-level associations. Spearman’s rank correlation coefficient was used to assess relationships between individual items, as well as between composite scores and selected outcomes. Correlation strength was interpreted based on standard classifications: r = 0.00–0.19 (very weak), 0.20–0.39 (weak), 0.40–0.59 (moderate), 0.60–0.79 (strong), and 0.80–1.00 (very strong) [15].

Binary logistic regression analysis was used to examine whether awareness and attitude scores (both as continuous and categorical variables) were significant predictors of referral behavior (Q18), interest in further education (Q19), and referral to orthodontists (Q20). Odds ratios [Exp(B)] and 95% confidence intervals were reported. A *p*-value of <0.05 was considered statistically significant.

## 3. Results

### 3.1. Demographic Characteristics and Descriptive Statistics

A total of 113 pediatricians participated in the survey, of which 110 completed all questions and were included in the analysis. Due to the use of this open online distribution strategy and the snowball sampling approach, the exact number of invited participants could not be determined; therefore, the response rate could not be calculated. Demographic data collected through Q1–Q5 are presented in Table 2. The majority of respondents were female (62/110, 56.4%). Most participants (37/110, 33.6%) had between 6 and 15 years of professional experience. Regarding workplace distribution, 41.8% (46/110) of participants were employed in public hospitals, followed by 32.7% working in private hospitals. More than half of the pediatricians (59/110, 53.6%) reported examining 31 or more patients per day.

Most pediatricians acknowledged an association between mouth breathing and maxillary constriction (Q9: 73.6%), although 26.4% reported insufficient knowledge regarding this relationship. Clinical experience was the most commonly cited source of information (Q10: 45.5%), while 28.2% stated that they had no knowledge of the topic. Awareness of maxillary expansion treatment was notably low (Q15: 14.5%); however, the majority believed it could have positive effects on both respiratory function (Q16: 68.2%) and facial aesthetics (Q17: 68.2%). Although only 0.9% of participants reported referring solely to orthodontists (Q20), 30% indicated that they refer to orthodontists in combination with other specialties. In addition, a significant proportion expressed a desire for further education in this area (Q19: 70.9%). A detailed summary of these findings is provided in Table 3.

### 3.2. Chi-Square Analysis of Relationships Between Demographic Variables and Questionnaire Responses

Chi-square analysis revealed several statistically significant associations between demographic variables and specific questionnaire items. These are summarized in Table 4. No other significant differences were found.

### 3.3. Reliability and Correlation Analysis of the Referral and Education Domain

Internal consistency analyses were performed for all subscales included in the questionnaire. Cronbach’s alpha values for the awareness (Q6–Q11) and attitude (Q12–Q17) subscales were found to be 0.658 and 0.787, respectively, indicating an acceptable level of reliability for both domains. However, Cronbach’s alpha for the subscale covering referral practices and educational interests (Q18–Q20) was −0.208, suggesting significant inconsistency within this group.

While 25.5% of participants (Q18) reported referring children with respiratory problems for orthodontic evaluation, 70.9% (Q19) indicated an interest in receiving additional training in this area. However, in item Q20, while only 0.9% of participants reported referring solely to orthodontists, 30% indicated that they refer to orthodontists in combination with other specialties. In contrast, 64.5% reported primarily referring to ENT specialists, 4.5% to allergy specialists, and 0.9% reported not referring at all.

A negative correlation was notably observed between items Q18 and Q20 (r = −0.27). When Q20 was removed from the analysis, Cronbach’s alpha increased to 0.155, revealing better conceptual consistency between Q18 and Q19. Thus, item Q20 represents a distinct construct and was, therefore, analyzed separately.

### 3.4. Descriptive Statistics of Awareness and Attitude Scores

The composite awareness score (Q6–Q11) had a mean of 4.65 (SD = 1.30), a median of 5.0, and a range from 0 to 6. A total of 67.3% of participants scored 5 or above, indicating high awareness.

The composite orthodontic treatment attitude score (Q12–Q17) had a mean of 4.03 (SD = 1.67), with a median of 5.0 and minimum and maximum values of 0 and 6. A total of 57.3% of participants demonstrated a high attitude level (score ≥ 5).

### 3.5. Spearman Correlation Analysis

Spearman’s rank correlation analysis was applied to assess the relationships between specific questionnaire items. The analysis revealed statistically significant positive correlations between some of the questions (Table S1). In particular, a strong positive correlation was observed between the source of information regarding the relationship between mouth breathing and maxillary constriction (Q10) and awareness of the problems that may result from maxillary constriction (Q11) (r = 0.685). Similarly, a moderate positive relationship was found between belief in the potential of orthodontic treatment to improve nasal breathing (Q13) and awareness of its effects on airway function (Q14) (r = 0.489). Another noteworthy finding was the very strong correlation between belief in the positive impact of maxillary expansion on facial aesthetics (Q17) and the tendency to refer children with respiratory problems for orthodontic evaluation (Q18) (r = 0.998). However, this exceptionally high correlation value should be interpreted with caution, as it may indicate conceptual overlap or item redundancy at the measurement level.

In addition to item-level Spearman correlation analyses, composite scores for awareness and attitude were also examined. The analysis revealed a moderate positive association between attitude scores and referral behavior (Q18), (r = 0.420), while awareness scores showed a weaker but still positive correlation (r = 0.333).

### 3.6. Logistic Regression Analysis

Binary logistic regression analyses indicated that pediatricians’ orthodontic treatment attitude scores were a significant predictor of both general referral for orthodontic evaluation (Q18) and referral specifically to orthodontists (Q20) (Table 5). The awareness score was not a significant predictor in any of the models. Neither awareness nor attitude scores predicted interest in further education (Q19).

## 4. Discussion

This study examined the relationship between pediatricians’ awareness of mouth breathing and related craniofacial anomalies and their referral behavior to orthodontists. Early diagnosis and referral are important to prevent complex anomalies and reduce treatment duration and costs. Importantly, while orthodontic treatment can play a valuable role in addressing anatomical and structural contributors to mouth breathing, it should be viewed as an adjunct to comprehensive care. The primary causes of mouth breathing—such as upper airway obstruction, allergic conditions, and dysfunctional breathing patterns—typically require medical management and functional therapies delivered through an interdisciplinary team [4,6,7]. To our knowledge, no previous study has systematically evaluated how pediatricians’ awareness and attitudes toward these multidisciplinary aspects influence referral patterns.

From an orthodontic standpoint, pediatricians appear to be generally aware of dentofacial anomalies; however, this awareness often does not lead to systematic screening or referrals. Koufatzidou et al. [12] reported that while pediatricians acknowledged the importance of oral examinations, many lacked the knowledge to perform orthodontic evaluations. Similarly, Dzaja and Tadin [13] found that although over 95% of pediatricians recognized the impact of malocclusion on quality of life, referral rates remained low due to a mismatch between knowledge and practice. In Türkiye, only 28.9% of pediatricians routinely assessed patients orthodontically, while 96.4% expressed a need for further training, and only 12.6% had received any [14]. These findings indicate that referrals are often symptom based rather than part of a structured assessment approach.

A major contributing factor is the lack of orthodontic training in pediatric education programs. Polek et al. [16] reported that an overwhelming majority of pediatricians expressed a desire for more education in this area. Sharma et al. [17] noted that only 45% had received relevant training during medical education and that referrals were more common among older physicians and those working in university hospitals. While clinical experience and institutional setting seem to support awareness and referrals, structural barriers such as time limitations and workload still pose significant challenges.

In the present study, demographic variables such as age, gender, years of professional experience, workplace setting, and patient load were recorded to provide a comprehensive overview of the participants’ backgrounds. Sharma et al. [17] found that referrals were more common among pediatricians over 35 years. Similarly, workplace type and patient volume can influence decision making. Public hospitals may limit thorough examinations due to workload, while private and university hospitals allow more time and resources. Our findings support these observations. Female pediatricians had higher awareness of the prevalence of mouth breathing (Q6), and those with more than 31 patients per day often cited clinical experience as their main source of knowledge (Q10).

Referral frequency was also significantly associated with workplace type (Q18). University hospital pediatricians reported more frequent referrals, possibly due to better access to information, interdisciplinary teams, and more time per patient. In contrast, referral rates were lower in public hospitals and more variable in private settings. These results highlight the influence of workplace conditions on awareness and referral practices.

### 4.1. Awareness of Mouth Breathing and Maxillary Constriction (Q6–Q11)

In this study, pediatricians were generally found to have a high level of awareness regarding the association between mouth breathing and maxillary constriction in children. Pediatricians’ knowledge advantage in this area may be attributed to their specialized training in child health and development, which includes topics on malocclusion and oral habits [12,14]. However, our results also showed that clinical experience, rather than formal education, was most often cited as the main source of knowledge on this topic.

Although both our findings and previous reports indicate that pediatricians and family physicians generally recognize key etiological factors for malocclusion—such as heredity, pacifier use, and thumb sucking—yet other contributors like nail biting and mouth breathing are less consistently acknowledged. This highlights the need for comprehensive education programs that address the full range of risk factors for malocclusion, not just the most common ones [14,18].

According to the Spearman correlation analysis, those who acquired their knowledge through clinical experience were more aware of the potential consequences of maxillary constriction (e.g., posterior crossbite and reduced arch length). Participants who believed that orthodontic treatment could improve nasal breathing also demonstrated higher awareness regarding airway development. These findings suggest that pediatricians perceive orthodontic interventions not only as aesthetic procedures but also as contributors to respiratory function. Similarly, Koletsi et al. [19] and Gómez-González et al. [20] reported that orthodontic and myofunctional approaches improved respiratory patterns and alleviated issues such as open bite and tongue thrust. Additionally, recent research by Kandasamy [7] supports the positive effects of early orthodontic intervention on nasal airflow and respiratory function.

Composite score analysis revealed that attitude toward orthodontic treatment was moderately correlated with referral behavior, while awareness showed a weaker positive correlation. This interpretation aligns with previous findings indicating that beliefs about the functional and aesthetic benefits of orthodontic interventions can guide referral practices. Studies have emphasized that mouth-breathing children often present with narrower airways and that early treatment—including myofunctional therapy—can improve facial development and airway patency [3,7,19,20].

### 4.2. Attitudes Toward Orthodontic Treatment (Q12–Q17)

Building on earlier findings [7,19,20], recent studies have further demonstrated that orthodontic and myofunctional therapies contribute not only to improved respiratory function but also to enhanced facial growth and airway patency in children with mouth breathing [21,22]. A similar level of understanding was evident among the pediatricians surveyed, with the majority (86.4%) acknowledging that orthodontic treatment influences skeletal development, including aspects such as facial aesthetics and jaw alignment. Additionally, 83.6% and 81.8% believed it could improve nasal breathing and airway function, respectively. Despite limited familiarity with maxillary expansion therapy (14.5%), 68.2% believed the intervention could positively influence both respiratory function and facial appearance, aligning with findings emphasizing its role in improving airway dimensions and posture [3,7].

Spearman correlation analysis revealed a moderate association between the belief that orthodontic treatment improves nasal breathing (Q13) and the perception that it benefits airway function (Q14). Likewise, those who perceived positive aesthetic effects of maxillary expansion (Q17) were more likely to refer patients for orthodontic evaluation (Q18), although this may reflect item overlap.

Beyond individual items, composite score analysis demonstrated that a more favorable clinical attitude toward orthodontic treatment was moderately correlated with referral behavior. These findings are consistent with the literature suggesting that while pediatricians may recognize the importance of malocclusion [16], actual referral behavior is more strongly influenced by clinical orientation toward orthodontic care. In contrast, neither awareness nor attitude scores were significantly associated with interest in further education (Q19), indicating that such intentions may be shaped by external factors unrelated to knowledge or beliefs. Modeling these outcomes using composite scores rather than individual items provided a clearer view of how broader clinical orientation translates into decision making.

### 4.3. Referral Practices and Educational Interest (Q18–Q20)

While just 25.5% of pediatricians indicated that they routinely refer children with mouth breathing to an orthodontist, a substantially higher proportion (70.9%) expressed a desire for further training. This highlights both an awareness of gaps in their current knowledge and a proactive attitude toward professional development. This is consistent with previous findings indicating that although pediatricians are generally interested in dental and orthodontic topics, they often lack structured clinical knowledge and training [11,12,13,14].

Multidisciplinary collaboration is essential for the effective management of mouth breathing due to its multifactorial impact on both medical and dental health [6]. Analysis of referral preferences revealed that while only 0.9% of pediatricians referred patients solely to orthodontists, 30% indicated that they include orthodontists as part of their multidisciplinary referrals. The majority, however, reported primarily referring to ENT specialists. This indicates a tendency to perceive mouth breathing predominantly as a medical issue, potentially overlooking its orthodontic implications. The recent literature highlights that although clinicians acknowledge the health risks associated with mouth breathing and sleep-disordered breathing, interdisciplinary referral pathways are still underutilized, often due to communication gaps and insufficient cross-disciplinary education [4].

Internal consistency analysis of the referral-related items (Q18–Q20) revealed poor reliability. Further analysis showed a negative correlation between referral frequency (Q18) and referral destination (Q20), suggesting conceptual divergence. As a result, Q20 was excluded from composite score modeling and interpreted independently. While some participants indicated they referred patients, these referrals were seldom directed toward orthodontists.

Composite-score-based logistic regression showed that a positive clinical attitude toward orthodontic treatment was a significant predictor of both general (Q18) and orthodontist-specific referrals (Q20), whereas awareness score was not significant in either model. Neither score predicted interest in further education (Q19).

Additionally, the number of years of clinical experience was significantly associated with referral behavior: pediatricians with more years in practice were more likely to refer patients for orthodontic evaluation. This suggests that awareness and confidence in making referrals may increase with cumulative clinical exposure, supporting prior findings in the literature [17].

Taken together, the findings offer partial support for the proposed hypothesis. While awareness alone did not significantly predict either general or orthodontist-specific referral behavior, a more favorable clinical attitude toward orthodontic treatment was strongly associated with an increased likelihood of both. This suggests that knowledge may be necessary but not sufficient; attitudinal orientation may play a more decisive role in translating awareness into clinical action.

Therefore, while orthodontic interventions can support anatomical remodeling and improve airway function, they must be integrated with medical management and functional therapies to comprehensively address the underlying causes of mouth breathing and promote correct breathing patterns.

### 4.4. Future Research Directions

Future initiatives may include the development and testing of structured e-learning modules to enhance pediatricians’ knowledge and screening skills regarding mouth breathing. Additionally, implementing pilot screening programs in pediatric clinical settings could help evaluate practical approaches to early identification and referral. Finally, establishing interdisciplinary diagnostic and management pathways through collaboration with ENT specialists and speech–language pathologists would contribute to more comprehensive and effective care for affected children.

## 5. Limitations

This study has several limitations that should be acknowledged. First, the voluntary nature of participation and the online survey format may have introduced selection bias, as pediatricians who are more interested in the topic might have been more likely to respond. Although participants were recruited from diverse regions across Türkiye, the sample may not fully represent all possible practice settings, institutional profiles, or geographic areas. Consequently, caution is warranted when generalizing the findings to all pediatricians nationwide or internationally.

Second, the self-reported nature of the data may have been affected by social desirability bias, potentially leading to overestimation of certain knowledge or attitudes. Additionally, the cross-sectional design of the study prevents any causal inferences regarding the relationships observed between awareness, attitudes, and referral practices.

Finally, while the questionnaire was developed based on a literature review and expert input and content validity was assessed through expert review, further psychometric validation—including factor analysis and test–retest reliability—was not performed. Future research employing validated instruments and longitudinal designs is needed to build upon these findings.

## 6. Conclusions

Based on this sample, pediatricians generally demonstrated high awareness of mouth breathing and its orthodontic implications. However, knowledge alone did not consistently translate into referral practices. Our results suggest that clinical attitude and conviction may play an important role in influencing referral behavior. Interest in professional development also appeared to be influenced by institutional and contextual factors, although this trend should be interpreted cautiously given the study’s cross-sectional design. Accordingly, educational strategies could be designed to not only enhance knowledge but also foster pediatricians’ confidence in the importance and benefits of orthodontic referral. Integrating structured orthodontic assessment and promoting interdisciplinary collaboration within pediatric training may help translate awareness into clinical action and contribute to improved patient outcomes.

## Figures and Tables

**Table 1:** Questions of survey besides the demographic questions.

**Awareness Regarding Mouth Breathing and Maxillary Constriction**	**Q 6:** Do you think mouth breathing is a common issue among children?	**Yes**
		**No**
		**I do not have enough knowledge**
	**Q 7:** Do you think that prolonged mouth breathing can negatively affect facial and jaw development in children?	**Yes**
		**No**
		**I do not have enough knowledge**
	**Q 8:** Do you think mouth breathing can have negative effects on facial aesthetics?	**Yes**
		**No**
		**I do not have enough knowledge**
	**Q 9:** Do you think maxillary constriction (narrow upper jaw) is more common in children who breathe through their mouths?	**Yes**
		**No**
		**I do not have enough knowledge**
	**Q 10:** From which source did you gain knowledge about the relationship between mouth breathing and maxillary constriction?	**Medical education**
		**Clinical experience**
		**Articles and books**
		**I do not have enough knowledge**
	**Q 11:** Were you previously aware of the problems caused by maxillary constriction (e.g., posterior crossbite, crowding due to reduced arch length, etc.)?	**Yes**
		**No**
		**I do not have enough knowledge**
**Attitudes Toward Orthodontic Treatment and Maxillary Expansion**	**Q 12:** Do you think orthodontic treatment, in addition to correcting dental crowding, also affects interjaw relationships and facial aesthetics?	**Yes**
		**No**
		**I do not have enough knowledge**
	**Q 13:** Do you think orthodontic treatment can improve nasal breathing and be effective in the treatment of mouth breathing in children?	**Yes**
		**No**
		**I do not have enough knowledge**
	**Q 14:** What positive effect can orthodontic treatment have on the airways of growing children?	**Upper airway expansion**
		**Lower airway expansion**
		**Has no effect on airways**
		**I do not have enough knowledge**
		**Upper and lower airway expansion**
	**Q 15:** Are you familiar with maxillary expansion treatment?	**Yes**
		**No**
		**I do not have enough knowledge**
	**Q 16:** Do you think maxillary expansion treatment in children has an effect on respiratory functions?	**Yes**
		**No**
		**I do not have enough knowledge**
	**Q 17:** Do you think maxillary expansion treatment in children can positively affect facial aesthetics?	**Yes**
		**No**
		**I do not have enough knowledge**
**Referral Practices and Educational Interest**	**Q 18:** Do you refer children with respiratory problems for an orthodontic evaluation?	**Often**
		**Rarely**
		**Never**
	**Q 19:** As a pediatrician, would you be interested in receiving additional training on orthodontic treatment for children with respiratory problems?	**Yes**
		**No**
		**I am not sure**
	**Q 20:** To which specialty do you most often refer children whom you suspect of having mouth breathing?	**Ear, nose, and throat specialist**
		**Allergy specialist**
		**Orthodontist**
		**All**
		**None**

**Table 2:** Demographic characteristics (Q1–Q5) of pediatricians who participated in the survey (*n* = 110).

Demographic Questions	Answers	Q1 (Gender)		Total (n = 110)
		Female (n = 62)	Male (n = 48)	
(Q2) Age	26–35 years	12 (80.0%)	3 (20.0%)	15 (13.6%)
	36–45 years	22 (71.0%)	9 (29.0%)	31 (28.2%)
	46–55 years	9 (42.9%)	12 (57.1%)	21 (19.1%)
	56 years and above	19 (44.2%)	24 (55.8%)	43 (39.1%)
(Q3) Years in practice	1–5 years	10 (83.3%)	2 (16.7%)	12 (10.9%)
	6–15 years	24 (64.9%)	13 (35.1%)	37 (33.6%)
	16–30 years	11 (44.0%)	14 (56.0%)	25 (22.7%)
	>30 years	17 (47.2%)	19 (52.8%)	36 (32.7%)
(Q4) Work sector	Public hospital	30 (65.2%)	16 (34.8%)	46 (41.8%)
	Private practice	3 (33.3%)	6 (66.7%)	9 (8.2%)
	Private hospital	16 (44.4%)	20 (55.6%)	36 (32.7%)
	University hospital	13 (68.4%)	6 (31.6%)	19 (17.3%)
(Q5) Patients per day	1–10	7 (58.3%)	5 (41.7%)	12 (10.9%)
	11–20	12 (46.2%)	14 (53.8%)	26 (23.6%)
	21–30	8 (61.5%)	5 (38.5%)	13 (11.8%)
	>30	35 (59.3%)	24 (40.7%)	59 (53.6%)
Total		62 (56.4%)	48 (43.6%)	110 (100%)

Q: question; *n*: number.

**Table 3:** Descriptive distribution of pediatricians’ responses to questionnaire items.

Question	Answers	Distribution of Answers (%)
Q 6	Yes	100 (%90.9)
	No	6 (%5)
	Not enough information	4 (%3.6)
Q 7	Yes	109 (%99.1)
	No	0
	Not enough information	1 (%0.9)
Q 8	Yes	105 (%95.5)
	No	0
	Not enough information	5 (%4.5)
Q 9	Yes	81 (%73.6)
	No	0
	Not enough information	29 (%26.4)
Q 10	Medical education	18 (%16.4)
	Clinical experience	50 (%45.5)
	Articles and books	11 (%10)
	Not enough information	31 (%28.2)
Q 11	Yes	37 (%33.6)
	No	40 (%36.4)
	Not enough information	33 (%30.0)
Q 12	Yes	95 (%86.4)
	No	2 (%1.8)
	Not enough information	13 (%11.8)
Q 13	Yes	92 (%83.6)
	No	2 (%1.8)
	Not enough information	16 (%14.5)
Q 14	Upper airway expansion	90 (%81.8)
	Lower airway expansion	1 (%0.9)
	Has no effect on airways	0
	Not enough information	19 (%17.3)
	Upper and lower airway expansion	0
Q 15	Yes	16 (%14.5)
	No	50 (%45.5)
	Not enough information	44 (%40.0)
Q 16	Yes	75 (%68.2)
	No	1 (%0.9)
	Not enough information	34 (%30.9)
Q 17	Yes	75 (68.2)
	No	0
	Not enough information	35 (%35.8)
Q 18	Often	28 (%25.5)
	Rarely	62 (%56.4)
	Never	20 (%18.2)
Q 19	Yes	78 (%70.9)
	No	15 (%13.6)
	Not sure	17 (%15.5)
Q 20	Ear, Nose, and Throat specialist (ENT)	71 (%64.5)
	Allergy specialist	5 (%4.5)
	Orthodontist	1 (%0.9)
	All	32 (%29.1)
	None	1 (%0.9)

Q: Question.

**Table 4:** Pairs of demographic and response variables with statistically significant associations. Only variable pairs with *p* < 0.05 based on Chi-square test results are included in the table.

	Variable 2	*p*-Value
Q1 (Gender)	Q6	0.043 *
Q2 (Age)	Q20	0.001 ***
Q3 (Years in practice)	Q20	0.004 **
Q4 (Patients per day)	Q10	0.024 *
Q5 (Work sector)	Q18	0.043 *

Q: question. * *p* < 0.05; ** *p* < 0.01, *** *p* < 0.001.

**Table 5:** Binary logistic regression results for each outcome (Q18: referral for orthodontic evaluation, Q19: interest in further education, and Q20: referral to orthodontists) based on awareness and orthodontic treatment attitude scores.

Question	Variable	Coef. (β)	Std. Error	Odds Ratio (Exp(β))	95% CI Lower	95% CI Upper	*p*-Value
Q18	Awareness score	0.45	0.29	1.57	0.89	2.77	0.19
Q18	Orthodontic treatment attitude score	0.90	0.31	2.46	1.33	4.57	0.001
Q19	Awareness score	0.09	0.22	1.09	0.70	1.68	0.69
Q19	Orthodontic treatment attitude score	0.13	0.14	1.13	0.86	1.49	0.37
Q20	Awareness score	0.11	0.24	1.12	0.7	1.79	0.64
Q20	Orthodontic treatment attitude score	0.44	0.19	1.56	1.08	2.25	0.02

Values are rounded to two decimal places unless otherwise indicated. Odds ratios (Exp(β)) and 95% confidence intervals are presented for each predictor. *p* < 0.05.

## Data Availability

The data collected and analyzed in this study are based on a questionnaire survey and include personal responses from pediatricians. Due to privacy and ethical considerations, the dataset is not publicly available. However, it can be provided by the corresponding author upon reasonable request and with appropriate ethical approval.

## Supplementary Materials

The following supporting information can be downloaded at: https://www.mdpi.com/article/10.3390/children12060790/s1, Table S1: Spearman correlation results.

## Abbreviations

The following abbreviations are used in this manuscript:

ENT	Ear, nose, and throat
Exp(B)	Exponentiated coefficient (odds ratio)
Q	Questionnaire item
SD	Standard deviation
SPSS	Statistical Package for the Social Sciences
