# Supplementary material for: Mouth Breathing and Orthodontic Referral in Pediatric Practice: A Cross-Sectional Survey
Source: Children (Basel). 2025 Jun 17;12(6):790. doi: 10.3390/children12060790 (PMC12192267; doi:10.3390/children12060790)
Supplement: Supplementary file 1 [file children-12-00790-s001.zip › children-3692159-supplementary.pdf]

Table S1. Spearman correlation results

	Years at work	Patients per day	Work Sector	Q6	Q7	Q8	Q9	Q 10	Q 11	Q 12	Q 13	Q14	Q15	Q 16	Q17	Q18	Q 19
Years at work																	
Patients per day	-.243*																
Work Sector	.258**	-.390**															
Q 6	-.051	-.253**	.094														
Q 7	-.077	.084	-.103	.290**													
Q 8	-.123	.192*	-.047	.077	.439**												
Q9	-.173	-.050	.098	.314**	.160	.266**											
Q 10	-.054	-.195*	.107	.256**	.127	.089	.685**										
Q 11	-.098	.086	-.028	.095	.123	.118	.337**	.323**									
Q12	-.028	.084	-.020	.064	.246**	.431**	.068	.098	.123								
Q 13	-.033	-.016	.008	.211*	.221*	.143	.133	.111	.235*	.249**							
Q14	-.051	-.048	.057	.184	.205*	.011	.204*	.308**	.226*	.226*	.489**						
Q 15	-.123	-.022	.080	.064	.109	.107	.279**	.355**	.415**	.282**	.144	.342**					
Q 16	-.099	-.052	-.008	.198*	.142	.229*	.261**	.353**	.264**	.246**	.492**	.450**	.253**				
Q 17	-.103	-.046	-.015	.195*	.140	.226*	.256**	.348**	.255**	.242*	.486**	.444**	.248**	.998**			
Q18	-.174	.100	-.172	.044	.014	.151	.320**	.307**	.310**	.169	.345**	.276**	.161	.437**	.435**		
Q 19	.014	.070	.021	.048	.115	.265**	.028	-.117	.126	.090	.165	.068	.165	.089	.085	.078	
Q 20	.202*	-.025	.059	-.056	.066	.059	-.084	-.108	-.157	.069	-.128	-.057	-.079	-.205*	-.208*	-.267**	.089

Q: Question.
*$p<0.5$, **$p<0.01$.
